# Green innovation and income inequality: A complex system analysis

**DOI:** 10.1016/j.strueco.2022.09.007

**Published:** 2022-12

**Authors:** Lorenzo Napolitano, Angelica Sbardella, Davide Consoli, Nicolò Barbieri, François Perruchas

**Affiliations:** a European Commission Joint Research Centre (JRC-Seville), Seville, Spain; b Enrico Fermi Center, Rome, Italy; c SOAS University of London, London, UK; d INGENIO (CSIC-UPV), Universitat Politècnica de València, Spain; e Department of Economics and Management, University of Ferrara, Italy; f SEEDS - Sustainability, Environmental Economics and Dynamics Studies, Italy

**Keywords:** Economic fitness and complexity, Environmental innovation, Income inequality

## Abstract

The objective of this paper is to analyse the relationship between income inequality and environmental innovation. To this end, we use the Economic Fitness and Complexity algorithm to compute an index of green inventive capacity in a panel of 57 countries over the period 1970–2010. The empirical analysis reveals that, on average, inequality is detrimental to countries’ capacity to develop complex green technologies. Using non-parametric methods we further articulate this general finding and uncover interesting non-linearities in the relationship between innovation and inequality.

## Highlights

• Innovation can contribute to mitigate climate-induced hazards.
• Countries most exposed to environmental degradation are often poor and more unequal.
• Economic complexity links countries’ development path and green inventive capacity.
• Rich countries develop complex green technologies irrespective of income inequality.
• Middle-income countries with low inequality develop complex green technologies.

## 1 Motivation and objectives

The objective of this paper is to explore empirically the relationship between income inequality and environmental innovation. The motivations for such an endeavour are manifold. These two global challenges stem from different sources and manifest themselves in different ways, but are arguably tightly connected. On the one hand, the threat of climate-induced hazards calls upon systemic changes that include, among other things, technological progress to accelerate the development or refinement of sustainable products and processes (Hoffert et al., 2002, Pacala and Socolow, 2004, Popp et al., 2010). One of the key analytical challenges in this debate concerns the identification of the circumstances that facilitate the green transition. On the other hand, empirical evidence indicates that, besides the widely-known negative distributional issues, income inequality can also hinder economic performance (see e.g. Forbes, 2000; Barro, 2000). An intriguing, and yet little explored, claim in this regard is that such a negative impact depends on which portion of the income distribution is most affected by inequality (Voitchovsky, 2005). Further, scholars have found evidence of a vicious circle whereby innovation – not specifically green – exacerbates inequality (see e.g. Aghion et al., 2018) in a way that ultimately undermines the incentives to search for and develop new technologies (Weinhold and Nair-Reichert, 2009). Moreover, income inequality and demand for environmental innovation exhibit similar patterns since countries that are likely to suffer the strongest negative effects of environmental degradation are also among the poorest (Mendelsohn et al., 2006; Bathiany et al., 2018), and often exhibit high levels of income inequality (Roberts, 2001). Last but not least, and in consideration of the above, our empirical analysis connects two current policy debates concerning, on the one hand, socio-economic barriers to the development of clean technology (Sovacool and Griffiths, 2020) and, on the other hand, synergies and trade-offs associated with structural features of climate justice (Baek and Gweisah, 2013; Chancel, 2020).

Accordingly, the present paper fills some gaps in the analysis of the intricate connections between inequality and green innovation. First, most research focuses on the factors that favour the emergence of environmental technology (see Barbieri et al., 2016 for a review) but neglects the barriers that may prevent, or slow down, green innovation, with just a few exceptions (e.g. Vona and Patriarca, 2011). The second gap is that green technologies are often treated as a homogeneous block, which contrasts with the huge diversity of goals they are designed for (Perruchas et al., 2020), with the breadth of forms of know-how involved (OECD, 2011) as well as the differential degree of maturity that each has achieved (Barbieri et al., 2020a). Such a high diversity is a peculiarity of the green technology domain (Barbieri et al., 2020b) and, obviously, affects the distribution of green innovation capacity across countries (Sbardella et al., 2018) or regions (Barbieri et al., 2022).

Bringing together these insights, we explore the relationship between domestic income inequality and capacity to generate green innovation in a panel of 57 countries over the period 1970–2010. To tackle these questions we propose a regression analysis on variables drawn from different sources. The dependent variable, green innovation capacity, is computed using the Economic Fitness and Complexity (EFC) algorithm on patent data (Tacchella et al., 2012; Cristelli et al., 2013). This recursive algorithm was originally designed to predict economic growth from country export data, and has been subsequently translated also to other domains, including innovation (Pugliese et al., 2019) and industrial sectors (Sbardella et al., 2017). The EFC algorithm has also been successfully applied to green innovation in a previous contribution (Barbieri et al., 2022, de Cunzo et al., 2022, Sbardella et al., 2018), while [30] applied the Economic Complexity Index approach (Hidalgo and Hausmann, 2009) to study green productive capabilities.

In a nutshell, the idea behind EFC is that the bipartite network that connects geographical areas to the outputs they produce (e.g. patents in the innovation domain, or exports in the trade domain) contains information about the existing local capabilities. For instance, the network that links countries to the (green and non-green) technologies in which they patent is informative about which technologies require the most advanced skills (i.e. have higher complexity), and which countries have the most advanced endowment of innovative capabilities (i.e. have higher fitness). By explicitly taking into account the complex structure of country-technology interactions, EFC enables us to differentiate countries not only according to the volume of green patents (as we would do by looking e.g. at patenting intensity) but also according to the composition of their patent portfolios. The main explanatory variable, income inequality, is built using information on net household income to calculate the Gini coefficient as well as various income percentile ratios. To address the second research question, we investigate whether the main result holds over the spectrum of green technologies (from the least to the most complex ones) and GDP.

The first key finding, based on parametric regression, is that income inequality exhibits significant negative correlation with country green technology fitness.^2^ On average, countries with high levels of inequality are also characterised by a lower level of green technology fitness. This entails that their technological capabilities connect with a narrower spectrum of green technological domains that are, also, less complex in relative terms. This holds for different measures of income inequality including the Gini coefficient and other income percentile ratios. The second key finding, based on non-parametric multivariate Nadaraya–Watson regressions (Nadaraya, 1964), is that the relationship between inequality and green fitness is non-linear and, crucially, depends on GDP per capita levels. This brings to the fore a number of important nuances with respect to the first general finding, in short: (i) high levels of inequality are especially detrimental for more complex green technologies; (ii) a certain amount of inequality appears to be necessary for the development of complex capabilities related to green technologies, especially in high income countries; (iii) low inequality opens up opportunities for countries with intermediate levels of per capita income to specialise in relatively complex green technologies; (iv) inequality is almost always associated with less complex technologies, the arena where upper–middle income countries are plausibly more proactive.

The contribution of our study are manifold. First, we enrich the scant literature on barriers to environmental innovations, by focusing on income inequality. Second, our metrics of complexity implicitly takes into account the heterogeneity associated with the exploration that precedes these technologies. This resonates with the rationale of policy interventions aimed at enhancing cross-fertilisation and boundary spanning across domains on know-how. In the present paper we discuss whether these actions need to be coherent with a more comprehensive economic policy that tackles inequality. This is also in line with the emerging discourse concerning the interdependencies between items of the Sustainable Development Goals agenda, wherein both environmental sustainability and inequality are prominent (e.g. Freistein and Mahlert, 2016). Third, combining parametric and non-parametric approaches adds important nuances to the analysis of the non-linear relationship between inequality and green technological capacity. Previous work (e.g. Vona and Patriarca, 2011) finds that the link between the development of green technologies and inequality is moderated by the level of per capita GDP. Our non-parametric approach confirms this and adds to it by providing new insights into the combination of GDP–income inequality that exhibits stronger association with more complex technological capabilities.

The remainder of the paper is structured as follows. Section 2 provides a review of the relevant literature followed in Section 3 by details on the main data sources and variable construction. Section 4 presents the empirical analysis while the last section concludes and summarises.

## 2 Theoretical background

### 2.1 The inequality–innovation nexus

Inequality and innovation are recurrent issues in the scholarly and policy domains, and so is their mutual relation. One strand of literature considers innovation as a determinant of inequality and emphasises the role of unbalances in the structure of labour markets and of wages. Technical change is known to have been traditionally biased in favour of more skilled workers and thus to accelerate the replacement of labour among the unskilled (Acemoglu, 2002) while skilled workers enjoy wage premia. The combination of these two processes has exacerbated inequality in several advanced economies, the US being one of the most widely documented cases (see e.g. Katz and Murphy, 1992). [4] contribute to this literature by looking at the distribution of income between labour and firm owners, and the rate of innovation. Their work provides theoretical and empirical support to the conjecture that innovation stimulates entrepreneurship but also increases income inequality. Indeed, the number of patents filed in US states is positively correlated to the top 1% income share. However, their results do not hold when broader measures of inequality are used, namely the Gini coefficient, Atkinson index, etc.

A second strand of literature investigates the extent to which inequality is a barrier for innovation. Here, the income distribution affects the development and diffusion of technologies via different channels. From a demand-side perspective, in a more equal society innovation becomes more attractive thanks to the incentive for the mass production of goods. [55] point out that, whereas the bottom and upper part of the distribution of income are more likely to demand, respectively, essential and customised products and services, middle class consumption generally concerns more standardised manufacturing goods, which strongly rely on incremental innovations. As observed by [48], greater equality triggers, among other things, the efficient use of resources, scale economies and, in particular, the rate of inventive activities. This implies that the mechanisms through which inequality affects innovation are best observed from a complementary supply side perspective. [29] suggest that a higher level of market participation by population brings about opportunities for innovation. Over the past decades, the improvement of old products and the re-organisation of production in the US agricultural and manufacturing sectors were favoured by the high degree of involvement of the middle class (Khan and Sokoloff, 2001). Moreover, broad market participation is linked to higher institutional quality – especially the protection of intellectual property rights – which made patenting simpler, cheaper and more accessible to US population in those years compared to other countries. The anecdotal evidence provided by these studies is confirmed by [55], who investigate the direct role of the middle class share on patenting activities. Their insights indicate that a more equal income distribution and strong intellectual property rights protection positively affects patent filing by residents.

The foregoing debate has rarely touched upon environmental innovation but there are grounds to believe that a broadly similar set of mechanisms are in place. The public good nature of environmental quality improvements entails that inequality is perceived as influencing innovative activities aimed at tackling local environmental problems, rather than global ones (e.g. urban PM10 reduction vs. global emissions). According to the demand-driven innovation approach, two main channels exist. The first is the so-called “pioneer consumer” effect whereby high-income consumers increase the demand for initially more expensive green products. In so doing they stimulate the production of this type of goods, thus leading to price reduction which, eventually, enables low-income consumers to add these products to their basket. The second effect is “consumption polarisation”, in which case excessive income difference between high and low income consumers reduces the potential externalities just mentioned. The seminal study by [54] points out that the relationship between inequality and the development of green innovative goods is highly non-linear: at low levels of per-capita income the pioneer consumer effect prevails, whereas at high level of per-capita income the reverse occurs. They also explore this empirical relation on a panel of OECD countries and find that inequality appears less detrimental for innovative activities at the beginning of their life-cycle, which is in line with the pioneer consumer effect.

Add to the above that recent policy debates call explicitly attention to synergies and trade-offs associated to the development context in relation to climate justice. Maximising synergies and avoiding trade-offs still entail significant challenges for developing countries, for vulnerable populations and for contexts with limited institutional, technological and financial capacity (Adger et al., 2003, Cappelli et al., 2021). Understanding the extent to which structural features such as inequality hamper capacity building has therefore become a guiding principle to assist the design of policy that tackles environmental and social justice priorities in a symbiotic, rather than detached, fashion (IEG, 2015, Vona, 2021).

### 2.2 The complexity of technological developments

The channels through which inequality affects innovation may lead to heterogeneous effects in relation to the type of technology under analysis (i.e. green vs. non-green) and/or the socio-economic conditions of the attendant countries (e.g. high, middle, low income countries). This research, however, neglects the characteristics of knowledge and how they moderate the inequality–innovation relationship. This gap is significant with regards to our understanding of green technologies. Using a set of patent-based indicators [11] find that green patents exhibit higher technological breadth, novelty and impact relative to non-green technologies. This implies higher costs and more uncertain knowledge recombination process which, in turn, leads to a potential underinvestment in their development, therefore calling for policy intervention to enable cross-fertilisation and boundary spanning. Further, a study on the diffusion of green technologies across countries by [46] emphasises that dealing with more complex portfolios of green technologies requires more advanced invention competences. From this it follows that, on average, high-income countries be endowed with more developed capabilities and thus be more likely major inventors of complex technologies. This resonates with recent findings on the spatial distribution of complex activities, taken as a whole and not necessarily related to specific (i.e. environmental) domains (Balland and Rigby, 2017).

In the present study, we investigate whether and to what extent the correlation between innovation and inequality varies according to the complexity of the country’s portfolio of green technologies. That is, we explore whether inequality represents a barrier to innovation in countries where the difficulty to produce different kinds of green knowledge is higher. By recalling the mechanisms depicted above, higher inequality provides less incentives to engage in more complex green innovative activities via a demand effect. In order to identify whether the pioneer consumer effect or the consumption polarisation mechanism dominates, it is crucial to account for the efforts spent in developing less and more complex green technologies. On the one hand, we expect inequality to be associated with less complex green technological capabilities due to the lower incentive to develop more complex technological solutions which require inventors to face higher cost and uncertainty in the knowledge generation process. On the other hand, inequality may represent a barrier also to more complex technologies. Such a relationship likely depends on the level of GDP. That is, a certain level of inequality may be necessary to provide the incentive to develop more complex capabilities, in line with the pioneer consumer effect. However, extremely high levels of inequality, especially in high income countries, may negatively affect the economic returns of more complex capabilities due to a lower demand effect.

## 3 Data and variable construction

For this study we create a panel of up to 57 countries for the period 1970–2010. The main variables measure green innovation capacity, income inequality and country characteristics. The choice of the time periods is contingent upon the availability of data, primarily on green patents and income inequality. We rely on a variety of sources that are described in greater detail below.

### 3.1 The economic fitness and complexity approach

The main dependent variable, country green technology fitness, is constructed by combining the raw patent data extracted from the PATSTAT database of the European Patent Office (EPO) with specific information about environment-friendly technologies collected by the OECD in the environment-related catalogue (ENV-TECH) (Haščič and Migotto, 2015). PATSTAT aggregates tens of millions of patent documents from over one hundred national and regional patent offices. They report, for each patent, the date of filing, the country of residence of inventors and applicants, the patent family (i.e. the group of patents that share the same priority filing and can be assumed to refer to the same invention), and a set of standard technology codes that classify the fields of technology in which the patent application introduced innovations with respect to the existing prior art at the time of filing. We exploit the ENV-TECH catalogue to collect patents that are relevant for green innovation. This allows us to partition the technological space at all levels of aggregation in a set of green classes and a set of non-green classes.

We exploit the recursive nature of the EFC algorithm, which defines the technological fitness of a country as a function of the complexity of the technologies in which it innovates and the complexity of a technology as a function of the fitness of the countries that produce them. In so doing we are able to consistently rank the elements of both sets and thus to tell more and less complex technologies apart. The EFC algorithm is part of a larger, and growing, literature based on the application of methodologies inspired by complexity science to a diverse array of empirical issues. These include macroeconomic forecasting (Hidalgo and Hausmann, 2009; Tacchella et al., 2018), the analysis of the evolution of the productive structures of nations (Hausmann et al., 2007; Hidalgo et al., 2007; Zaccaria et al., 2014), the relation between complexity and inequality (Hartmann et al., 2017, Sbardella et al., 2017), the assessment of capability accumulation and the study of interactions between capabilities in shaping knowledge creation as well as technological progress (Pugliese et al., 2017b, Napolitano et al., 2018). Indeed, it has been shown that technological capabilities are generally *nested* Napolitano et al., 2018, Pugliese et al., 2019; this implies that countries with a very specialised R&D output will mostly innovate in more ubiquitous (and hence mundane) technological fields.^3^ To illustrate, imagine a breakthrough in human mobility determined by electric self-driving vehicles (henceforth, technology Z). Let us assume that the above hinges around two key technologies: effective machine learning algorithms (tech. X) and reliable energy-dense batteries (tech. Y). From the EFC perspective, Z is more complex than its building blocks, X and Y, if the set of countries that innovate in Z is smaller than the set of countries that innovate in X or Y.^4^ This makes sense intuitively, since one might expect Z to be developed with higher probability in countries that also have capabilities to innovate in X and Y.

**Fig. 1:**
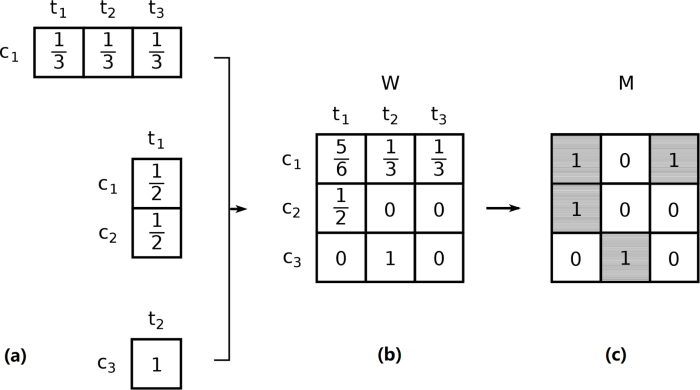
Construction of a binary data matrix for period y. Panel (a): each patent family is attributed a unit of weight, which is equally split among all combinations of inventor countries and technological codes included in patent applications belonging to the family which were filed during period y. Panel (b): a weighted matrix (W) is built in which rows correspond to countries and columns correspond to technology codes; every (country, technology) pair is attributed the corresponding sum of patent family shares. Panel (c): W is binarized creating matrix M, which is then fed to the EFC algorithm.

**Fig. 2:**
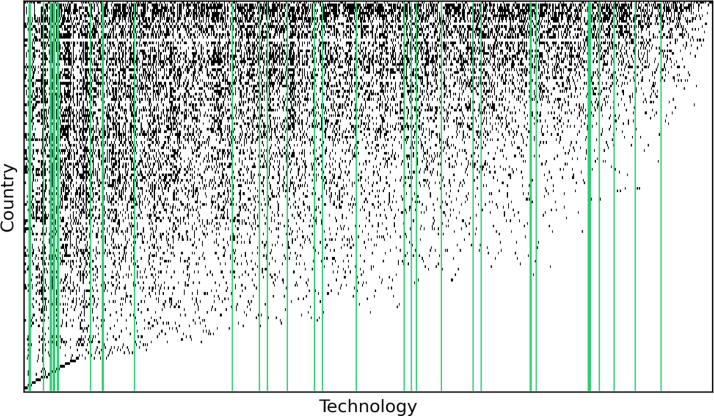
Country-Technology matrix ($M_{c,p}$) relative to the 2008–2012 time window. The rows and columns are ordered using the EFC algorithm. Each row corresponds to one of the 145 countries for which we have patent data, with black dots highlighting the technological areas to which each country is linked; fitness decreases from top to bottom. Each column represents a technology field; complexity increases from left to right. The matrix accounts for the full technological spectrum, i.e. all 692 green and non-green technology codes included in the analysis. The vertical green lines highlight the position in the complexity ranking of green technologies as defined by the 36 2-digit ENV-Tech codes listed in Table A.1. (For interpretation of the references to colour in this figure legend, the reader is referred to the web version of this article.)

**Fig. 3:**
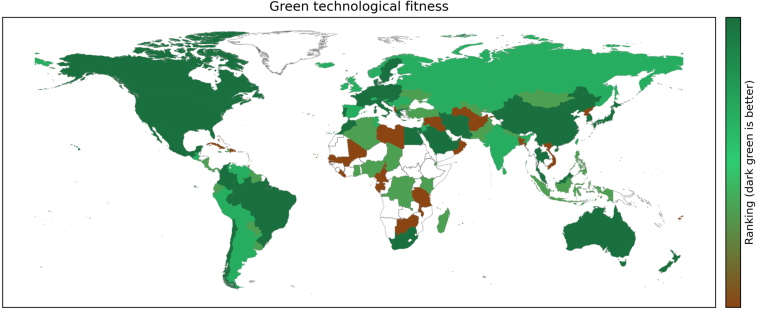
Snapshot of the geographic distribution of the Green Fitness Ranking across countries. Colour coding: dark green (top of the ranking), light green (middle) to brown (bottom). White: no data available. (For interpretation of the references to colour in this figure legend, the reader is referred to the web version of this article.)

**Fig. 4:**
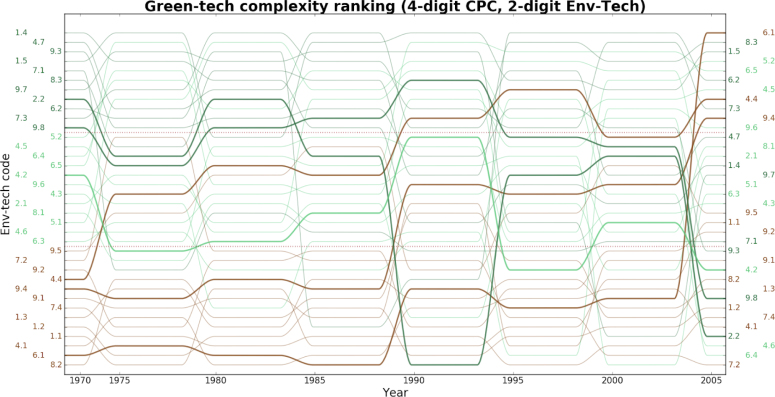
Time evolution of the Complexity Ranking of green technologies (5-year time-windows). Higher ranked (more complex) technologies at the top. (For interpretation of the references to colour in this figure legend, the reader is referred to the web version of this article.)

**Fig. 5:**
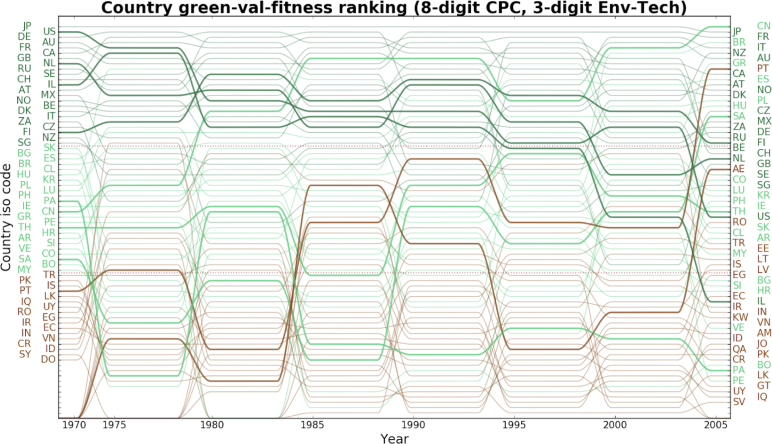
Time evolution of the green fitness of countries (5-year time-windows). Higher ranked (higher fitness) countries at the top. (For interpretation of the references to colour in this figure legend, the reader is referred to the web version of this article.)

**Fig. 6:**
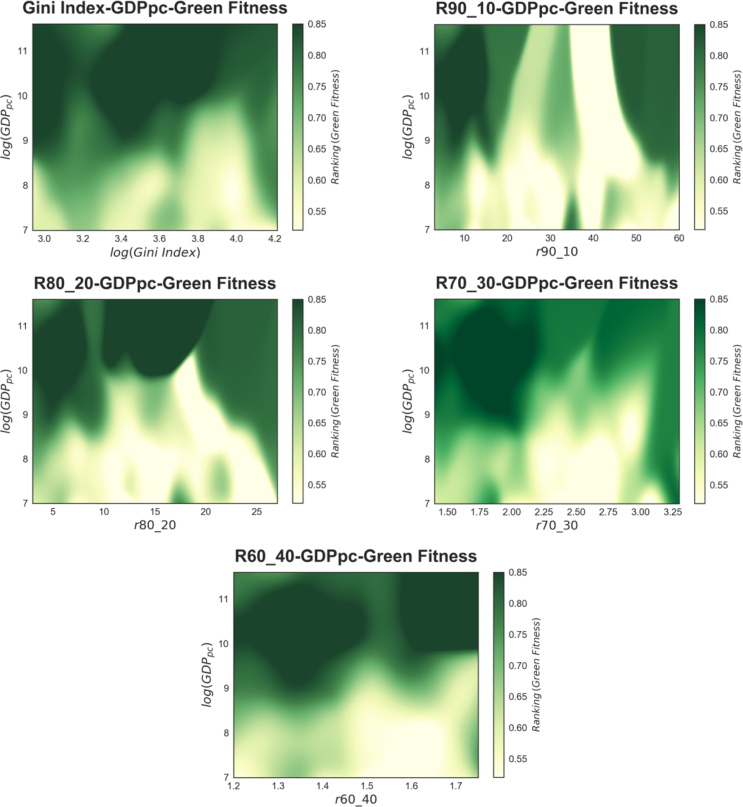
The three-dimensional relation between different measures of income inequality – namely the logarithm of Gini index, and the 90:10, 80:20, 70:30 and 60:40 income decile ratios – on the x-axis (5-year time-windows), the logarithm of GDP per capita on the y-axis, and the ranking of green fitness on the z-axis (5-year time-windows). Each colour map represents the expected value of green fitness given the income inequality measure and GDP per capita and is obtained with a non-parametric Nadaraya–Watson kernel estimation by pooling all countries and years in our database. (For interpretation of the references to colour in this figure legend, the reader is referred to the web version of this article.)

**Fig. 7:**
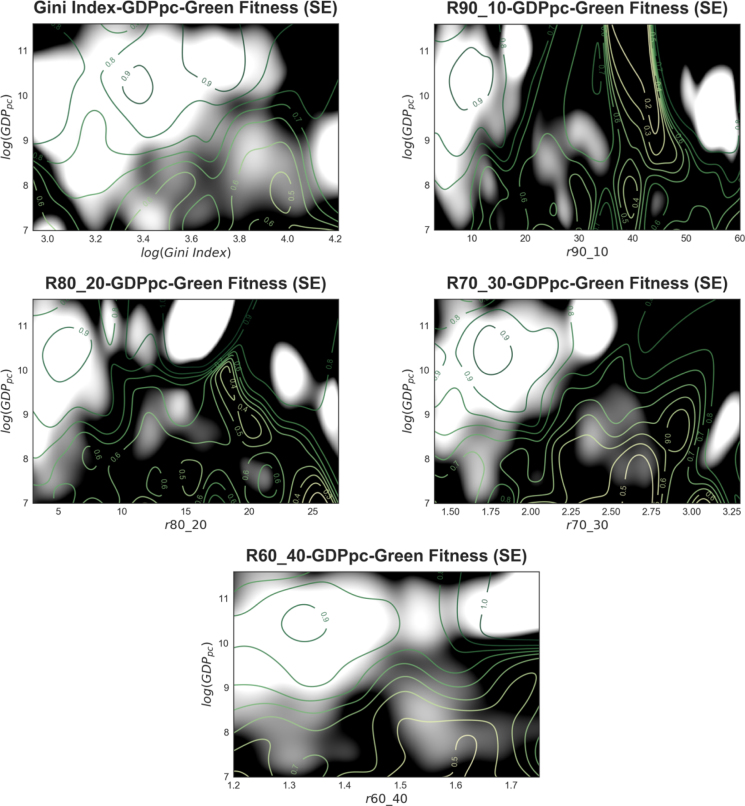
Estimation errors of the green fitness colour-maps in Fig. 6. The plots are built with the same data as Fig. 6. Two layers of information are represented in this figure. (1) In the black and white scale, the standard error of the green fitness ranking mean estimated through the Nadaraya–Watson regression with Gaussian kernel. White indicates a standard error of 2% or less, and black a standard error of 4% or more. (2) In the green shades, the iso-lines of the green fitness ranking levels (lowest in light green, highest in dark green). The plot is obtained by pooling all countries and years comprising our database. (For interpretation of the references to colour in this figure legend, the reader is referred to the web version of this article.)

**Fig. 8:**
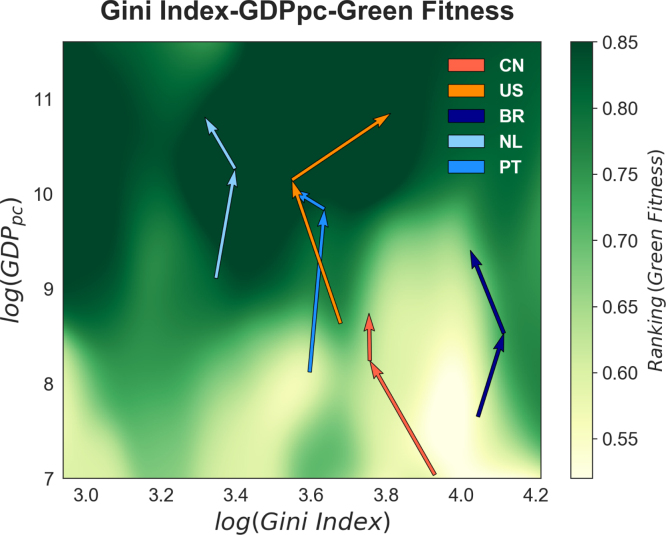
Gini index colour map with country trajectories. We superimpose on Fig. 6 the trajectories of Brazil, China, Portugal, the Netherlands, and the US in the Gini index-GDP per capita plane. The arrows point in the direction of time and show the starting, middle and final year for which the country in question presents a Gini index observation (1981, 1998, 2012 for Brazil, 2002, 2008, 2012 for China, 1977, 1996, 2012 for the Netherlands, 1980, 2005, 2011 for Portugal and 1971, 1992, 2012 for the US), where their x-values represent, when possible, 5-year rolling averages of the Gini index. (For interpretation of the references to colour in this figure legend, the reader is referred to the web version of this article.)

As mentioned in Section 1, to apply the EFC algorithm to our data and obtain a measure of the fitness of countries, we need to represent the countries and the technologies in which they innovate at a given point in time as a bipartite graph. To this aim, we assume that every patent family is an invention and assign it one unit of weight. For each year, we split active patent families equally among all combinations of technology code and inventor country that contribute to it. This way, we obtain a measure of the observed innovation intensity of each country in each technological field that we summarise in a weighted matrix W (see Fig. 1 for an example). We then binarize the weighted matrix W filtering it through the index of Revealed Comparative Advantage (Balassa, 1965)^5^ and obtain M such that:

(1) $$M_{c,t}\left(y\right)=\left\{\begin{array}{cc} 1\; & \text{if }\frac{W_{c,t}}{\underset{t^{\prime }}{\sum }W_{c,t^{\prime }}}>\frac{\underset{c^{\prime }}{\sum }W_{c^{\prime },t}}{\underset{c^{\prime },t^{\prime }}{\sum }Wc^{\prime },t^{\prime }}\\ 0\; & \text{otherwise.} \end{array}\right)$$

The binary matrix M, which links countries to the technologies in which they produce more than their *fair share* of patents, is then fed to the EFC algorithm, which is defined as follows:

(2) $$\left\{\begin{array}{cc} {\overset{˜}{F}}_{c}^{\left(n\right)}=\underset{t}{\sum }M_{c,t}Q_{t}^{\left(n-1\right)}\text{ , }\; & Q_{t}^{\left(n\right)}=\frac{{\overset{˜}{Q}}_{t}^{\left(n\right)}}{\langle {\overset{˜}{Q}}_{t}^{\left(n\right)}\rangle }\\ {\overset{˜}{Q}}_{t}^{\left(n\right)}=\frac{1}{\underset{c}{\sum }M_{c,t}\frac{1}{F_{c}^{\left(n\right)}}}\text{ , }\; & F_{c}^{\left(n\right)}=\frac{{\overset{˜}{F}}_{c}^{\left(n\right)}}{\langle {\overset{˜}{F}}_{c}^{\left(n\right)}\rangle } \end{array}\right)$$

with initial condition:

(3) $$\underset{t}{\sum }Q_{t}^{\left(0\right)}=1\;\forall t.$$

The key outputs of the recursive EFC algorithm are the Fitness of countries (F) and the complexity of technologies (Q), which are simultaneously determined by the network connections encoded in the input matrix, M. It is worth noting that, unlike e.g. trade, patenting activity does not produce a regular flow of output. Hence, yearly patenting counts can be rather noisy, especially at higher levels of granularity. This can produce fluctuations in the binary country-technology networks which are not linked to dynamics of the underlying capability structure. For this reason, in our analysis we define $W_{t}$ on a 5-year time window comprising the closed interval [t,t+4]. This allows striking a good balance between noise and number of observations To clarify, $W_{t}\left(c,t\right)$ is the average patenting by country t in technology t over the time window. This choice carries over to $M_{t}$ and to the Fitness and Complexity rankings. The Fitness of a country ($F_{c}$) is the sum of the complexity of the outputs to which it is linked,^6^ while the Complexity of a technology ($Q_{t}$) is a non-linear function of the fitness values of the countries that include that technology in their baskets. Non-linearity plays a crucial role in this context because the main term in defining the complexity of a technological field is the least fit country that innovates in that field. In other words, the Complexity of any technology is limited by the lowest country Fitness that enters its computation.^7^ The underlying rationale is that the fitness of a country captures the competitive advantage that the underlying endowment of capabilities accrues. The more diverse the set of capabilities, the more complex is the technology space available to the country. Conversely, a country with low fitness has less capabilities and, therefore, is limited to less complex technological domains. Depending on the structure of the input matrix, the EFC algorithm is known (Pugliese et al., 2016) to converge to zero fitness and zero complexity at different speeds for different countries and technologies respectively. However, this is not an issue because it is always possible to define a consistent ranking along both dimensions. For this reason, our main dependent variable is the fitness ranking and not the fitness scores of countries. The same is true for technological complexities, which we employ to tell more complex technologies apart from less complex ones in the second part of the empirical analysis.

Fig. 2 displays a binary country technology-matrix, in which rows and columns have been ordered using the EFC algorithm. Rows represent countries and are arranged by decreasing fitness from top to bottom, while columns are ordered to reflect increasing technological complexity from left to right. The columns in the matrix represent the full spectrum of CPC and ENV-TECH codes available in the data, and the ordering of rows reflects technological fitness, not green fitness. The matrix thus ordered displays a characteristic nested structure in which higher fitness countries tend to be more diversified and lower fitness countries are linked to a subset of the fields to which higher-fitness countries are connected (see Tacchella et al., 2012 for country-product matrices and Sbardella et al., 2017 for region-industrial sector matrices displaying a similar nested structure). Consequently, the probability of having a connection with rare technological fields, i.e. those which require the most advanced capabilities, increases as fitness increases.

Once we have computed the complexity of all technologies, and hence also the Technological Fitness of countries, via Eq. (2), we can also compute their Green Technological Fitness. To this aim, we apply the fitness formula only to the ENV-TECH codes (highlighted by the vertical green lines); this way associate each country to the complexity of all the technologies to which it is linked.^8^ The vertical green lines highlight the position in the complexity ranking of all green technologies, which are spread out over the entire spectrum. This means that both low- and high-complexity green technologies exist.

Last but not least, Fig. 3 shows the distribution of inventive efforts across countries as captured by the fitness ranking of countries. This broad brush picture indicates that environmental innovative activities are relatively more intense (brighter green) in North America, Europe, Russia, China, Japan, and Australia, while countries in Latin America and in the Middle East are in the mid to bottom part of the ranking (brown to red).

### 3.2 Income inequality and other dimensions of interest

The main explanatory variable in our regression analysis is income inequality. To build this, we retrieve information on household net income from the World Income Inequality Database (WIID),^9^ which covers most of the countries over the period 1867–2012. For the purposes of the present paper, we compute five measures of income inequality for each country. The first is the Gini coefficient, a widely used proxy of inequality with wide geographical data coverage. Since a major goal of this paper is to look into differences between various portions of the income distribution, we also extract from the WIID information on deciles of disposable income, and compute the following ratios: ninth to first decile (90:10); eighth to second (80:20); seventh to third (70:30); sixth to fourth (60:40). We envisage that this ample selection of variables affords a more nuanced understanding of the extent of different levels of inequality across the board.

To account for country characteristics, we include a battery of variables into our empirical analysis, namely: GDP per capita (source: World Bank^10^), the percentage of population with tertiary literacy (source: Barro-Lee^11^) and population density (source: World Bank.^12^.) To ensure coherence across the data, we build averages over five-year intervals of all the variables under analysis. While this is dictated by the format of the data on literacy, which is only available at that frequency, taking averages over periods allows us to account for the fact that green innovation, inequality, and the other dimensions under analysis change slowly over time.

## 4 Analysis

### 4.1 Economic fitness, green innovation and inequality

Fig. 4 provides details about the evolution over time of the ranking of green technologies based on their complexity. To ensure consistency with the rest of the empirical analysis, the rankings are computed over 5-year time windows, with the first observation covering the period 1970–1974 and the last observation covering the 2005–2009 window. Technological complexity decreases from top to bottom. The left and right y-axes report the complexity rankings relative to the first and last time window respectively. For ease of visualisation, the y-labels and plot lines are coloured to reflect their position in the oldest ranking. The mix of the label colours along the right y-axis shows that the complexity of technologies has varied substantially over the time period.

In the upper third of the ranking are domains that have maintained their leadership, namely *Enabling Technologies for GHG Mitigation in Waste Water Treatment and Waste Management* [8.3], *Environmental Monitoring* [1.5], and *Rail Transport* [6.2], as well as technological fields that have caught up, in particular *Road Transport Technologies* [6.1], *Nuclear Energy* [4.4], and *Technologies for the processing of minerals* (used in industrial production, e.g. cement, glass) [9.4].^13^ All these instances of technologies that have worked their way up the ranking speak to the extent to which new and advanced capabilities have kept pouring into the attendant fields since the 1970s. In contrast to these, there are domains that have lost prominence, for example *Supply-Side Technologies for Water Availability* [2.2], or *Enabling Technologies for GHG Mitigation in the Production or Processing of Goods* [9.8]. Further notice that, on the one hand, transitions from low- to high complexity can be rather abrupt, as in the case of *Road Transport Technologies* [6.1] while, on the other hand, movement along the ranking need not be monotonic as shown by *Supply-Side Technologies for Water Availability* [2.2] and *Energy Generation From Fuels Of Non-Fossil Origin* [4.2], which display a highly variable degree of complexity. On the whole, this ranking resonates with empirical studies on the life cycle of green technology, whereby established fields like *Renewable Energy Generation* [4.1] are in the bottom third reflecting how mature the attendant knowledge base is and, relatedly, how ubiquitous is that technology. In contrast, *Capture/Disposal of GHG other than Carbon Dioxide* [5.2] is still at relatively early stage of development, and thus exhibits higher complexity and less ubiquity (see Barbieri et al., 2020b).

Fig. 5 builds on the former technology ranking and plots the time trajectory of all the countries included in the analysis along the fitness ranking. Similar to Fig. 4, each observation refers to 5-year intervals starting in 1970, country labels are coloured according to the corresponding fitness ranking in the first window and countries with higher fitness are displayed at the top. Notice that, contrary to technologies, country ranking positions are more stable and that where they take place changes in ranking are less abrupt. This is intuitively plausible considering that while imitation in mature fields of technologies can lead to quick catching up by capability-poor countries, the global set of capabilities that defines a country’s technological reach is the result of a long-term accumulation process, which can therefore generate some inertia in the fitness values and the relative performance. Two notable exceptions in our sample are Portugal (PT), the United Arab Emirates (AE) and Panama (PA), all of which display relatively high variability in the central time periods with the first two leaping to the top all at once between 2000–2004 and 2005–2009 and the latter stabilising at the bottom. Further notice that the relative stability of the green fitness ranking does not mean that no long-term trends can be observed. For example, Israel (IL) and the United States of America (US) start high up in the ranking and constantly drop, while China (CN) gradually reaches the top. Note in passing that some lower fitness countries are not always present in the plot, but appear only when their first green patents are recorded.

**Table 1:** Parametric regression results — Green technology fitness.

	(1)	(2)	(3)	(4)	(5)
*Gini (ln)*	−8.748**				
	(3.629)				
*90:10*		−0.126**			
		(0.0492)			
*80:20*			−0.424***		
			(0.125)		
*70:30*				−4.143	
				(3.192)	
*60:40*					−7.929
					(8.894)
*GDP pc*	2.956***	2.920***	2.892***	2.791***	3.012***
	(0.698)	(0.888)	(0.860)	(0.962)	(0.915)
*Pop Density*	−0.0607	0.00424	−0.0158	−0.0182	−0.0493
	(0.0756)	(0.205)	(0.185)	(0.203)	(0.198)
*Schooling*	0.0736	−0.0413	0.0616	0.00539	0.0712
	(0.134)	(0.212)	(0.125)	(0.222)	(0.161)
*Country FE*	Yes	Yes	Yes	Yes	Yes
*Time trends*	Yes	Yes	Yes	Yes	Yes

$R^{2}$	0.593	0.614	0.632	0.601	0.606
Obs.	273	199	207	198	207

Notes: Dependent variable is mean Green Technology Fitness per country per year. Country fixed effects (FE) and country-specific time trends are included in the model. Robust standard errors in parenthesis. * p<.1, ** p<.05, *** p<.01.

**Table 2:** Parametric regression results — Total technology fitness.

	(1)	(2)	(3)	(4)	(5)
*Gini (ln)*	−6.074				
	(4.784)				
*90:10*		−0.0423			
		(0.0579)			
*80:20*			−0.135		
			(0.143)		
*70:30*				4.393	
				(5.650)	
*60:40*					9.383
					(12.67)
*GDP pc*	−0.582	0.800*	0.601*	1.282	0.789*
	(1.051)	(0.459)	(0.334)	(0.908)	(0.458)
*Pop Density*	1.153	−1.012	−0.982	−1.081	−1.012
	(1.015)	(0.944)	(0.917)	(1.010)	(0.948)
*Schooling*	−0.592	0.322	0.215	0.345	0.149
	(0.503)	(0.415)	(0.306)	(0.444)	(0.281)
					
Country FE	Yes	Yes	Yes	Yes	Yes
Time trends	Yes	Yes	Yes	Yes	Yes

$R^{2}$	0.184	0.618	0.621	0.620	0.622
Obs.	280	204	213	203	213

Notes: Dependent variable is mean total technology Fitness per country per year. Country fixed effects (FE) and country-specific time trends are included in the model. Robust standard errors in parenthesis. * p<.1, ** p<.05, *** p<.01

### 4.2 Parametric approach

In this section, we explore the relationship between environmental-related technological complexity and income distribution by means of parametric regression analysis. The empirical strategy investigates whether there is a significant correlation between countries’ green fitness and income inequality over the period 1970–2010. We estimate the following empirical model:

$$GreenFitness_{it}=\alpha +\beta Inequality_{it}^{A}+\gamma X_{it}+\sigma_{i}+\tau_{it}+{ɛ}_{it}$$

where the dependent variable, GreenFitness, is country green technological fitness, described in Section 3. Further, we articulate the relationship between country green fitness and income inequality by assessing whether the results hold for the entire spectrum of technologies or the finding is peculiar to green technologies. The main independent variable is within-country income distribution (Inequality) measured using alternative indicators (A) such as the Gini coefficient or the decile ratios 90:10, 80:20, 70:30 and 60:40. As mentioned, given the low pace at which inequality evolves over time (Quah, 2001), we employ 5-year time windows. The model we estimate also includes country fixed effects (σ) and a set of controls such as population density, per capita GDP, and a measure of schooling (i.e. the percentage of population with tertiary literacy) (X). Finally, country-specific time trends (τ) are included to control for unobservable heterogeneity that varies over time in each country.^14^

Table 1 shows the results of the model estimation. The main finding is that there is a negative and significant association between a country’s income inequality and green fitness. This means that countries with higher levels of inequality reduce the incentives to develop more complex capabilities related to green technology. According to the literature, higher complexity is associated with higher costs and uncertainty in the development of new technologies. In this context, inequality plays a pivotal role by exacerbating the expected returns from innovative activities. Such an association is confirmed when we use the Gini index and the income distribution ratios 90:10 and 80:20 as a proxy for income inequality. It is worth noting that this result takes into account the intensity of the inventive activities in each technological domain and the complexity of technological fields. In addition, the results show a positive correlation of the green fitness variable with a proxy for human capital (i.e., Schooling) and a negative association with population density — although these two variables are not statistically significant. Finally, GDP per capital shows a positive and significant correlation with green technology fitness: as expected richer countries perform better in terms of investments in environmental-related R&D.

Further, we explore whether this association holds also when taking into account fitness computed on the entire technological spectrum instead of only on green technologies. Table 2 shows that the coefficients of the inequality measures are not statistically significant when we consider all the technologies developed in a specific country. This suggests that inequality may act as a barrier especially when green technological capabilities are concerned, and that this effect might be due to the higher heterogeneity of the knowledge base of this group of technologies (Barbieri et al., 2020a). However, non-linearity may also affect these findings. In the next section we explore these patterns through a non-parametric approach that enables us to observe this relationship at each level of inequality, green fitness, and GDP per capita.

### 4.3 Non-parametric approach

In this section, we explore the relationship between inequality and green technology fitness using a non-parametric approach. Fig. 6 depicts a graphical tool to visualise qualitatively the joint relation between green technological capabilities as proxied by green fitness, GDP per capita, and the proposed measures of income inequality (Gini coefficient, the 90:10, 80:20, 70:30 and 60:40 income decile ratios), where the variables employed are built in the same fashion as in our parametric approach presented in Section 4.2. In particular, each panel of Fig. 6 represents a colour-map of the relation between income inequality on the x-axis, the logarithm of GDP per capita on the y-axis, and a non-parametric estimate of the green fitness ranking on the z-axis. The values of the latter are captured by different shades of green such that the darker the green the higher the fitness. To build this figure, we pool all the countries and years in our panel and take 5-year moving averages of the aforementioned variables to ensure coherence across the data. The colour-maps are obtained via a multivariate Nadaraya–Watson regression (Nadaraya, 1964), a continuous non-parametric method, with a Gaussian kernel. In practice, we estimate the conditional expected value of the dependent variable, the green fitness ranking, given the independent variables, GDP per capita and income inequality, by calculating locally weighted averages of the green fitness ranking, where the weights are Gaussian kernels.

Fig. 7 shows the standard errors of the Nadaraya–Watson means. Herein, the darker areas correspond to a standard error of 4% or above, while the white ones to a standard error of 2% or less. To allow comparability, the iso-levels of the green fitness ranking estimations are superimposed on the plots in Fig. 6. In the upper portion of the plot, irrespective of their income inequality level, countries with high levels of per-capita income display generally high levels of green fitness. This is not surprising considering that high-income countries are more likely endowed with more developed capabilities, therefore income inequality is likely to constitute less of a barrier to producing knowledge in complex technologies.

An interesting feature of the plots in Fig. 6 is the diagonal movement of colour, which hints at an interplay between income inequality and GDP per-capita in contributing to the green fitness ranking. The figures suggest that there is a threshold of GDP per-capita below which it is unlikely that a country will be able to develop a sufficient number of complex technologies to obtain high green fitness. Countries located in the upper left corners of the plots in Fig. 6 are characterised by high GDP per capita and low income inequality; they are therefore expected to achieve the highest positions in the green fitness ranking. Low income inequality lowers such threshold and allows also countries with intermediate levels of per capita income to develop the capabilities necessary to increase their capacity to innovate in relatively complex green technologies and thus achieve intermediate levels of green fitness.

Put otherwise, a country’s wealth might not be a barrier to developing advanced competencies for environmental innovation insofar as income inequality is not too high. This offers a qualitative hint at the fact that a more equal distribution of income matters for unleashing innovation capacity among both low- and mid-level income countries. Hence, high income inequality appears to be a barrier for innovation capacity among both low- and mid-level income countries. Notice that the evidence is consistent when we take as measures of inequality the intermediate income decile ratios, the top and bottom ratios, and the Gini coefficient. The only apparent exception is the 90:10 ratio case; however, looking at the standard error of the estimation in Fig. 5, we see that the significant regions of the green fitness ranking estimation are placed only in the left and in the upper–middle quadrants of the r90:10-$log\left(GDP_{p}c\right)$ plane, where the colour pattern is consistent with that of the other inequality measures. This suggests that the dark green region in the upper right portion of the 90:10 graphs is mostly an artefact of the non-parametric estimation technique and is not populated by actual data points as we do not find in our data-set countries with high levels of per capita GDP and intermediate 90:10 ratio values.

To provide some context on these maps, we superimpose on the basic terrain of Fig. 6 the trajectories of Brazil, China, Portugal, the Netherlands, and the US (Fig. 8). These trajectories are built selecting three points in time – the starting, middle and final year for which the country in question presents an observation for the Gini index – and each is composed of two arrows pointing in the direction of time. As the World Income Inequality Database contains a large number of missing points, especially for emerging and developing economies, the three years represented for each country are different. In particular for Brazil we consider the triplet (1981, 1998, 2012), for China (2002, 2008, 2012), for the Netherlands (1977, 1996, 2012), for Portugal (1980, 2005, 2011) and for the US (1971, 1992, 2012). The x-values of the arrows are built using 5-year rolling averages of the Gini index, with the exception of China, for which, given the short time-span and the few observations provided between 2002 and 2012, a 2-year rolling average is considered. Not only China presents fragmented observations, but also the most problematic point is its Gini index starting observation year, that unfortunately does not allow us to appreciate in its entirety China’s increasing Green Fitness trajectory — starting at the middle of the ranking at the end of the 1970s and reaching the top with a 0.9 value in 2012 –, as by 2002 it had started to climb the Green Fitness ladder and already showed a high position in the ranking. This is not clearly visible in the plot, the colour map is in fact the result of a non parametric estimation and the colour distribution is determined by localised averages of the points present in a small region of the plot and China, being an unicum, displays high Green Fitness, but GDPpc and Gini index values comparable to those of different lower Green Fitness countries. Therefore, while when analysing the movement of countries the Fitness-GDP plane, as is customary in the Economic Complexity literature (Cristelli et al., 2013, Tacchella et al., 2012), it is possible to observe the diagonal trajectory of China, whose higher fitness than per capita GDP has been interpreted as a source of growth potential and possibility for a “lateral escape from the poverty trap” (Pugliese et al., 2017a), in the Gini index-GDP per capita we observe here, the red arrow has it final point in an intermediate–high fitness region.

Having said this, by allowing to observe the simultaneous effect of income per capita and inequality on green fitness, this plot complements the descriptive analysis of the country rankings in the previous subsection, where the performance in green innovation of emerging and advanced economies has been commented on. As expected, the combined growth of income per capita and the decrease of inequality from the starting to the final point in time lead to an increase in green fitness. Only the US (in orange) departs from this behaviour: while remaining among the top green innovators, its Gini index increases over time. Indeed, for the US, which has already developed a very advanced set of capabilities and is able to produce the most complex green technologies, inequality does not seem to have a detrimental effect on innovative capacity.

## 5 Concluding remarks and the way ahead

The objective of this paper was to explore the empirical associations between income inequality and environmental innovation. These two global challenges exhibit similar incidence across space, whereby countries that are most exposed to the perils of environmental degradation are also among the poorest, and often suffer high levels of income inequality. Innovation, it has been argued, is the other main channel that links together environmental sustainability and inequality. Empirical evidence indicates that progress in technology is a key tool, albeit not the only one, to preserve the environment while maintaining high levels of economic performance. However, research has also convincingly demonstrated that innovation can be a trigger of inequality which, eventually, may undermine the ability to develop new technologies. Closer insights into how inequality and the environment interact can inform policymakers and other stakeholders involved in the design and the implementation of sustainable development.

While most literature insists on the factors that facilitate the emergence of new technology, we focus on the little-explored issue of country specific circumstances that act as barriers to the pursuit of environmental innovation. In particular, we focus on the knowledge bases of countries to explore the extent to which domestic technological capabilities rely on complex green technologies. In so doing, we acknowledge the heterogeneity of green technology as regards both the domains of know-how and of application. To this end, we relied on economic complexity approaches to account for the diverse nature of technological specialisation and for how this distributes across different institutional domains like countries. In addition, the paper contributes to the debate on the linearity of the relationship between inequality and technological development. To do so, we employed both parametric and non-parametric approaches to delve deeper into this relationship at different levels of inequality and GDP per capita.

Taking advantage of the nested structure of green patenting data we apply a complexity-based measure, the EFC algorithm, to define a fitness measure of the green technological competitiveness of each country. Such an approach affords the opportunity to study the green technology portfolios of each country, and therefore to look beyond measuring patenting intensity in isolation. In so doing we gain detailed information on the global structure of country-technology interactions and are able to explore the qualitative composition of inventive activities. Moreover, this method is consistent with the notion that innovating in any domain requires broad ensembles of specific and generic know-how as well as the ability to recombine these inputs. In short, innovation capacity goes hand in hand with a country’s long-term development path.

Of course, our analysis does not exhaust the potential applications of complexity-based measures to relevant questions in the field of innovation. In fact, our patent-based green fitness offers a rich yet synthetic measure of the degree of technological development of each country, and leaves interesting questions open to investigation, such as whether countries with similar degrees of fitness tend to follow similar paths over time and whether the mix of technologies observed in a country at a given point in time lead preferentially towards the expansion in a well-defined set of new fields. Additionally, based on the export specialisation profiles of countries, the EFC metric has already proven to be a valid tool in inferring a country’s manufacturing capabilities and in linking its potential of growth with its productive structure. Therefore, our measure of green technological fitness can provide additional information on the trajectories of national innovation systems, and, if put into relation with the export fitness, it could provide an even more nuanced representation of growth and development possibilities.

The empirical analysis yields two main findings. First, income inequality exhibits significant negative correlation with countries’ green innovation capacity. This suggests that, on average, the development of more complex green technological capabilities is mainly concentrated in countries with low inequality. A possible explanation can be extrapolated from the literature on the determinants of green innovation. On the one hand, higher complexity entails higher costs and uncertainty in the knowledge generation process that characterises exploratory activities. On the other hand, high inequality is detrimental to innovation since the externalities from the rich, pioneer consumers are lower. Combining these two aspects, we point out that the unequal distribution of income lowers the benefits arising from the development of complex technological capabilities. By exploring this relationship through a non-parametric approach, we shed light on the non-linear relationship between inequality and innovation. Our conjecture is that this relationship differs according to the levels of inequality and the countries’ wealth.

The second finding stems from the non-parametric approach: for high income countries, low levels of inequality are associated with higher capabilities in the development of more complex green technologies. In addition, increasing the level of inequality does not appear a barrier to green technology development. Rather, moderate levels of inequality facilitate specialisation in more complex technological fields. This is ascribed to the pioneer consumer effect. Further, low income inequality makes it possible also for countries with intermediate levels of per capita income to develop the capabilities necessary to increase their capacity to innovate in relatively complex green technologies. This indicates that a more equal distribution of income matters especially for low- and mid-level income countries where income inequality emerges as a barrier to green innovation capacity.

These findings bear relevance for policy insofar as the recent assessment of mitigation and adaptation strategies by the Intergovernmental Panel on Climate Change (IPCC, 2022) emphasises – together with the traditional roles of international cooperation, finance and innovation – also significant cross-country heterogeneity in climate vulnerability and in capacity building. The consensus is that policy that aims at supporting low-emission technology and business practices can only be effective if designed with a clear understanding of the attendant barriers, especially in developing countries (Adenle et al., 2015). Comprehensive instrument design that is consistent with national circumstances can support the shift towards equitable a low-emission future. The present paper calls attention to inequality as a burden for climate governance in those settings and, thereby, as a plausible root for renowned weak enabling conditions for investments in technology and competence development. Inequality is in fact often associated with inadequate and uncertain institutional settings characterised by lack of empowerment, high levels of informality, and adverse power dynamics that constrain the mainstreaming of climate action (Najam, 2005, Ramos-Mejía et al., 2018).

We conclude by emphasising that the issues at hand are indeed complex and that the limitations of the present analysis are a compass for further research. First, the recurrent caveat in innovation studies: we have only considered inventions that are captured by patents and, while technology is touted to be a major driver of the transition to sustainable economies, it is certainly not the only one. Future research could build on our effort at mapping innovation capabilities and develop more specific narratives of the manifold transformations that are at play. Second, climate change is a global phenomenon with local manifestations, and regional or city-level variation is crucial. Third, we account for domestic capabilities only indirectly, and do not delve into the skills-innovation nexus as mediated by the institutional dynamics of the attendant labour markets. Fourth, we have not explicitly accounted for trade as a channel for green technology diffusion. Finally, we focused on income inequality but remain aware of the manifold forms of inequality that matter for environmental issues. Unequal access to environmental goods, different degrees of exposure and vulnerability to environmental risks, and uneven effects of environmental policies are other important, if hard to measure, forms. While we are aware of these limitations, we also hope that the present paper will inspire future research on this compelling agenda.

**Table A.1:** 1 & 2-digit ENV-TECH codes and labels.

Code	1-Digit Class Description	2-Digit Class Description
1	Environmental Management	
1.1		Air pollution abatement
1.2		Water pollution abatement
1.3		Waste management
1.4		Soil remediation
1.5		Environmental monitoring

2	Water-related adaptation technologies	

2.1		Demand-side technologies (water conservation)
2.2		Supply side technologies (water availability)

4	CCMTs related to energy generation, transmission or distribution	

4.1		Renewable energy generation
4.2		Energy generation from fuels of non-fossil origin
4.3		Combustion technologies with mitigation potential (e.g., Using fossil fuels, biomass, waste, etc.)
4.4		Nuclear energy
4.5		Efficiency in electrical power generation, transmission or distribution
4.6		Enabling technologies in energy sector
4.7		Other energy conversion or management systems reducing GHG emissions

5	Capture, storage, sequestration or disposal of greenhouse gases	

5.1		$CO_{2}$ capture or storage (CCS)
5.2		Capture or disposal of greenhouse gases other than carbon dioxide ($N_{2}O$, $CH_{4}$, PFC, HFC, $SF_{6}$)

6	CCMTs related to transportation	

6.1		Road transport
6.2		Rail transport
6.3		Air transport
6.4		Maritime or waterways transport
6.5		Enabling technologies in transport

7	CCMTs related to buildings	

7.1	Integration of renewable energy sources in buildings	
7.2		Energy efficiency in buildings
7.3		Architectural or constructional elements improving the thermal performance of buildings
7.4		Enabling technologies in buildings

8	CCMTs related to waste water treatment or waste management	

8.1		Wastewater treatment
8.2		Solid waste management
8.3		Enabling technologies or technologies with a potential or indirect contribution to GHG mitigation

9	CCMTs in the production or processing of goods	

9.1		Technologies related to metal processing
9.2		Technologies relating to chemical industry
9.3		Technologies relating to oil refining and petrochemical industry
9.4		Technologies relating to the processing of minerals
9.5		Technologies relating to agriculture, livestock or agroalimentary industries
9.6		Technologies in the production process for final industrial or consumer products
9.7		Climate change mitigation technologies for sector-wide applications
9.8		Enabling technologies with a potential contribution to GHG emissions mitigation

## CRediT authorship contribution statement

**Lorenzo Napolitano:** Conceptualization, Methodology, Analysis, Writing. **Angelica Sbardella:** Conceptualization, Methodology, Analysis, Writing. **Davide Consoli:** Conceptualization, Methodology, Analysis, Writing. **Nicolò Barbieri:** Conceptualization, Methodology, Analysis, Writing. **François Perruchas:** Conceptualization, Methodology, Analysis, Writing.

## Figures and Tables

**Fig. B.1:**
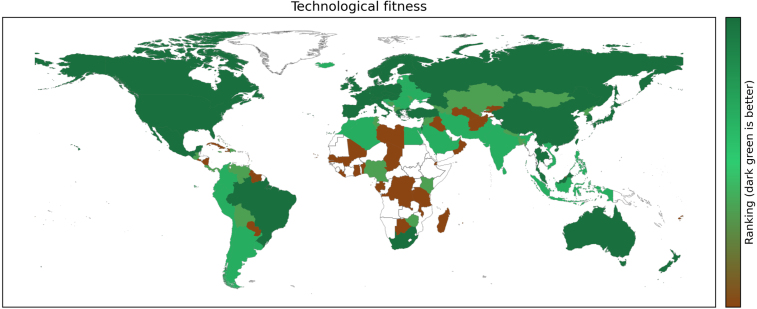
Snapshot of the geographic distribution of the Technological Fitness Ranking across countries. Colour coding: dark green (top of the ranking), light green (middle) to brown (bottom). White: no data available. (For interpretation of the references to colour in this figure legend, the reader is referred to the web version of this article.)

**Fig. B.2:**
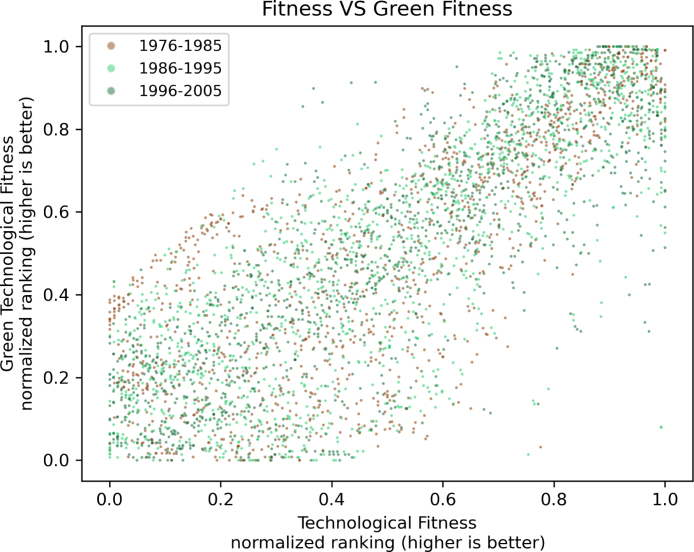
Technological Fitness ranking (x-axis) VS Green Technological Fitness ranking of countries. Rankings are normalised between 0 and 1 (with 1 being the best ranking). Dots refer to the ranking of a country at a specific point in time. Dot colours reflect the time window to which each observation belongs. Recall that rankings are computed based on average patenting over 5-year time windows; hence, for instance, a dot tagged as corresponding to year 2005 covers the period 2005–2009. The plot shows a positive association between Technological Fitness and GTF throughout the period under analysis. However, there is also considerable variability.

**Fig. C.1:**
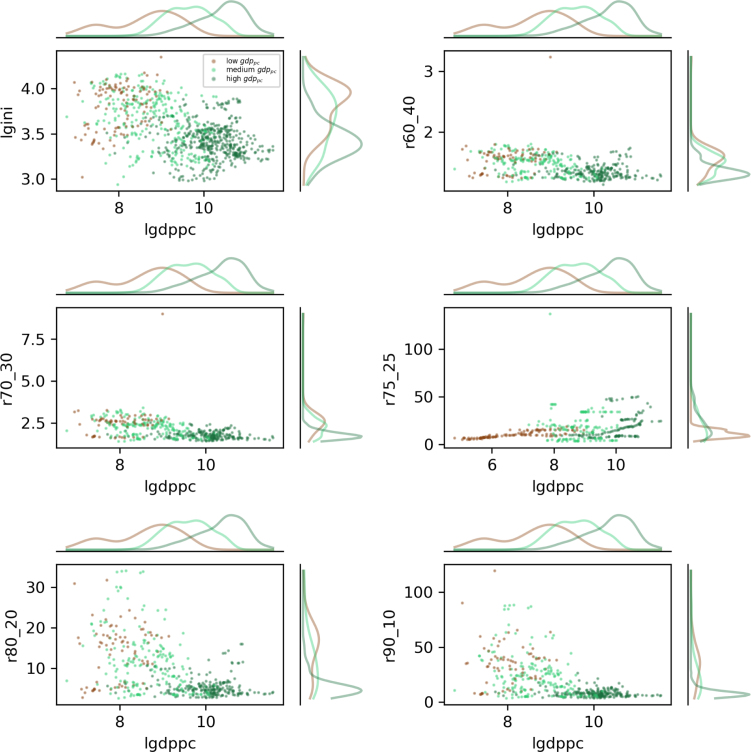
Pairwise joint distribution of $log\left(gdp_{pc}\right)$ with all inequality measures. The brown curves reflect observations relative countries in the bottom third of the distribution of average $gdp_{pc}$; the light green curves refer to countries with middle average $log\left(gdp_{pc}\right)$; the dark green curves refer to countries with high average $log\left(gdp_{pc}\right)$. (For interpretation of the references to colour in this figure legend, the reader is referred to the web version of this article.)

**Table A.2:** Parametric regression results — Green technology fitness.

	(1)	(2)	(3)	(4)	(5)
*Gini (ln)*	−8.748**				
	(0.779)				
*90:10*		−0.0640***			
		(0.0171)			
*80:20*			−0.160***		
			(0.0455)		
*70:30*				−1.260***	
				(0.393)	
*60:40*					−4.209***
					(1.088)
*GDP pc*	−0.683***	−0.571***	−0.532***	−0.479***	−0.454***
	(0.125)	(0.148)	(0.147)	(0.137)	(0.134)
*Pop Density*	−0.000309	0.0000373	−0.000438	0.000107	−0.000403
	(0.000445)	(0.000158)	(0.000415)	(0.000144)	(0.000435)
*Schooling*	−0.0190	−0.114**	−0.122***	−0.125**	−0.120**
	(0.0377)	(0.0531)	(0.0469)	(0.0551)	(0.0467)
*Country FE*	No	No	No	No	No
*Time trends*	No	No	No	No	No

R2	0.137	0.139	0.165	0.124	0.148
N	273	199	207	198	207

Notes: Dependent variable is mean Green Technology Fitness per country per year. Robust standard errors in parenthesis. * p<.1, ** p<.05, *** p<.01

## Appendix A ENV-TECH technologies

See Table A.1.

## Appendix B Technological fitness

In this section, we delve deeper in the description of the Technological Fitness of countries and its relation to GTF. The aim is to provide the interested reader with a bit more context and allow a qualitative comparison between these two measures, which are closely related and capture complementary aspects of the innovative capabilities of nations.

Fig. B.1 plots on a map the Technology Fitness ranking of countries, similarly to what Fig. 3 does for GTF. Comparing the two figures shows that the coverage is similar, meaning that most countries that patent, also file same patents in green technologies. Also the colour pattern is broadly similar, suggesting that there is a positive correlation between Technological fitness and GTF. Fig. B.2 further confirms the existence of a positive relation between Technological Fitness and GTF by plotting the ranking of countries at different points in time along the two dimensions against one another. The scatter plot shows that countries with a good Technological Fitness ranking also tend to rank well in terms of GTF. However, there is considerable dispersion, meaning that there are countries that rank high in terms of Technological Fitness that have low GTF and *vice versa*. The dots in the plot are coloured to reflect the time period to which they correspond. The colour pattern in the figure suggests that the association between the two measures does not depend strongly on time.

## Appendix C Joint distribution

See Fig. C.1.

## Appendix D OLS estimation

See Table A.2.

## Footnotes

2 A negative relationship between a measure of complexity and income inequality was previously obtained by [20]; however here we focus on the green innovative competitiveness of countries, and employ the EFC algorithm applied to green technological fields rather than the ECI index (Hidalgo and Hausmann, 2009) based on product-level export data.

3 A corollary is that countries that are able to advance knowledge in more complex (*rare*) fields, and thus achieve higher fitness, are able to innovate across the technological spectrum.

4 Notice that nothing is said about the intricacy of the inventions that rely on technology Z. In fact, a modern internal combustion engine has a far greater number of moving parts and components than an electric engine. Nevertheless, electric car technology could be more complex if less countries are well-equipped to develop it.

5 The sparsity of the data matrices ensures that results are robust to the choice of the binarization strategy.

6 Notice that we can meaningfully apply the same definition to a subset of the outputs. To clarify: the complexity of all technologies connected to a country determine its Technological Fitness; and the complexity of the Green Technologies linked to a country determine its Green Fitness.

7 It is worth noting that, depending on how Fitness is computed, it can be more or less sensitive to the country size (in economic terms). Indeed, we can in principle define an extensive measure of Fitness (employing W instead of M in Eq. (2)). However, employing M (which is binary and, more importantly, filtered via Revealed Comparative Advantage) allows to compute the ore conventional intensive Fitness metric, which allows to substantially reduce the effect of country size. In a nutshell, though larger economies produce higher volumes in general, their size will work against them when it comes to determining their fair share in a specific sector. Moreover, Cristelli et al., 2013, Tacchella et al., 2012 showed that computing Fitness via M produces a metric that correlates well with GDP *per capita*, while computing Fitness via W yields a metric that correlates well with GDP. In this paper we employ the intensive Fitness metric, which is far less sensitive to the effect of country size.

8 Notice that this reflects an *exogenous* approach to Green Technological Fitness, whereby we take into account the full technological spectrum and establish the complexity of green technologies also relative to non-green technologies. This implies that when we compute GTF from the complexity of the subset technologies that are tagged as green, we are not employing all the information available in M. Consequently, if we ordered the rows of M based on GTF, the footprint of complexity in the technological space defined by M would be less evident. To produce a nested matrix employing an ordering based on GTF, we would need to take an *endogenous* approach to green fitness like [34] and define M as the adjacency matrix of the network linking countries only to green technologies. Though valid in general, the endogenous definition would make the comparison between technological fitness and GTF less direct. For this reason, the exogenous approach is more adequate for our purposes. We thank an anonymous referee for prompting us to clarify this point about highlighting the nested structure of M via the complexity algorithm.

9 https://www.wider.unu.edu/project/wiid-world-income-inequality-database (Last access: 28 January 2019).

10 https://data.worldbank.org/indicator/NY.GDP.MKTP.CD (Last access: 28 January 2019).

11 http://www.barrolee.com/data/full1.htm (Last access: 28 January 2019)

12 https://data.worldbank.org/indicator/EN.POP.DNST (Last access: 28 January 2019)

13 The full list of ENV-TECH Green Technologies codes and labels is available on Table A.1 in Appendix A.

14 Table A.2 in Appendix D we also show the results of a model estimation which does not include country fixed effects and time trends. In this way results are comparable with the following analysis that investigates the relationship between inequality and green fitness in a non-parametric way.
